# Nanopore Sequencing in Veterinary Pathogen Detection: A Review of Technologies and Applications

**DOI:** 10.3390/vetsci13030216

**Published:** 2026-02-25

**Authors:** Lei Xu, Leilei Zhao, Zeyu Tong, Kai Peng, Mianzhi Wang, Runsheng Li, Zhiqiang Wang, Ruichao Li

**Affiliations:** 1 Jiangsu Co-Innovation Center for Prevention and Control of Important Animal Infectious Diseases and Zoonoses, Yangzhou University, Yangzhou 225009, China; 009063@yzu.edu.cn (L.X.);; 2 College of Veterinary Medicine, Yangzhou University, Yangzhou 225009, China; 3 Department of Precision Diagnostic and Therapeutic Technology, City University of Hong Kong Shenzhen Futian Research Institute, Shenzhen 518057, China; 4 Department of Infectious Diseases and Public Health, Jockey Club College of Veterinary Medicine and Life Sciences, City University of Hong Kong, Hong Kong 999077, China; 5 Tung Biomedical Sciences Centre, City University of Hong Kong, Hong Kong 999077, China; 6 Jiangsu Interdisciplinary Center for Zoonoses and Biosafety, Yangzhou University, Yangzhou 225009, China; 7 Jiangsu Key Laboratory of Zoonosis, Yangzhou University, Yangzhou 225009, China

**Keywords:** nanopore sequencing, veterinary medicine, diagnostics, clinical applications and challenges

## Abstract

Nanopore-based sequencing has emerged as a revolutionary tool for animal pathogen genomics, offering capabilities unattainable with Sanger and next-generation sequencing (NGS). Despite rapid technical progress, routine veterinary deployment still faces uncertainty in study design, sample preparation, and interpretation thresholds across diverse hosts and sample matrices. Accordingly, this review consolidates recent evidence and provides workflow-oriented guidance for veterinary diagnostics and One Health surveillance. Its portability, ability to generate real-time long-read data, and minimal infrastructure requirements enable rapid, on-site sequencing for veterinary diagnostics and surveillance. This review examines the principles of nanopore sequencing and its advantages over conventional methods, surveying recent applications across viral, bacterial (including antimicrobial resistance, AMR), and parasitic pathogen detection in animals. In viral diagnostics, it facilitates rapid whole-genome sequencing and outbreak tracing in field settings. For bacterial pathogens, it enables near-complete genome assembly and identification of plasmid-borne AMR genes. Emerging studies also demonstrate its utility in parasitology, from high-resolution species identification to whole-genome assemblies. We compare these advancements with traditional diagnostics, highlighting strengths in speed and comprehensiveness while addressing current limitations in accuracy and host-DNA interference. As technology matures through improvements in chemistry and adaptive sampling, nanopore sequencing is poised to transform veterinary pathogen detection and bolster One Health surveillance of emerging zoonoses.

## Simple Summary

Rapid and reliable pathogen detection is critical for animal health, outbreak response, and One Health surveillance. Nanopore sequencing is a portable long-read technology that can generate sequence data in real time, enabling flexible diagnostic strategies ranging from untargeted metagenomics to targeted amplicon sequencing and isolate whole-genome sequencing. In this review, we summarize how nanopore sequencing is being applied to detect viral, bacterial (including antimicrobial resistance), and parasitic pathogens in veterinary settings, and we compare its strengths and limitations with conventional methods such as PCR, culture, microscopy, and serology. We highlight practical workflow considerations from sample processing to data interpretation, discuss where nanopore sequencing can provide added value, and outline current barriers to routine implementation, including standardization, analytical thresholds, biosafety, and regulatory acceptance. We conclude with perspectives on how ongoing improvements in chemistry, basecalling, and enrichment strategies may support more consistent and standardized use in veterinary diagnostics and surveillance.

## 1. Introduction

The advent of third-generation sequencing technologies has revolutionized genomic analysis in the life sciences. Numerous nanopore sequencing platforms have already been increasingly used in veterinary medicine, including those developed by Oxford Nanopore Technologies (ONT), MGI CycloneSeq, and Qitan [1,2]. Unlike Sanger sequencing or next-generation sequencing (NGS, e.g., Illumina), nanopore sequencing directly reads long nucleotide strands in real time, without the need for fluorescent labeling or synthesis-based detection [3]. This fundamental shift confers several unique advantages: real-time data generation, portability of sequencers, and ultralong read lengths, all of which are highly valuable for rapid pathogen diagnostics and genome assembly. Indeed, the ability to perform field-portable, real-time long-read sequencing makes nanopore technology revolutionary for veterinary health applications.

Advances in nanopore sequencing offer major benefits for animal health and disease control. Many pathogens that threaten livestock, wildlife, and public health are difficult to diagnose quickly with traditional methods, which often require lab facilities and can only detect known targets. Nanopore sequencing, by contrast, can identify and characterize a wide range of viruses, bacteria, and parasites in a single run. Metagenomic ONT sequencing, for example, can reveal multiple pathogens and co-infections directly from clinical samples. Compact devices also allow testing in the field with minimal equipment, greatly shortening the time between sampling and diagnosis. This rapid, on-site capability is especially important for managing fast-spreading or emerging animal diseases [4].

**Figure 1:**
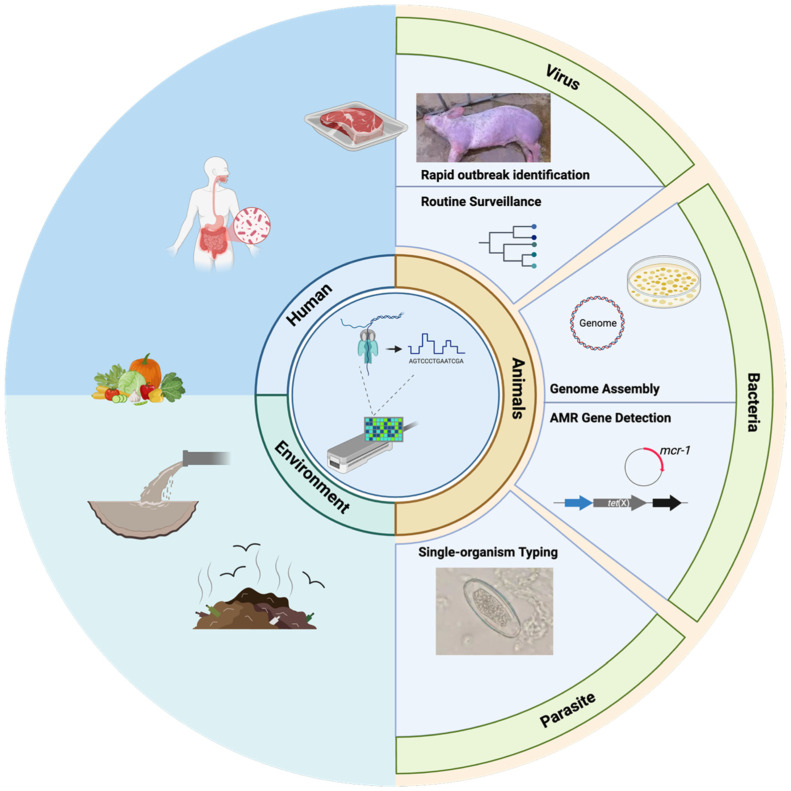
Overview of nanopore sequencing applications for animal pathogen detection within a One Health framework. The schematic illustrates how nanopore sequencing supports veterinary infectious disease investigations across animals, humans, and the environment. Applications include rapid outbreak identification and surveillance of viral pathogens, genome assembly and antimicrobial resistance (AMR) gene detection in bacteria, and high-resolution typing of parasitic pathogens. Animals are shown as the central focus, highlighting the role of nanopore sequencing in veterinary diagnostics and One Health surveillance.

In most veterinary workflows, nanopore sequencing is currently used to complement PCR-based diagnostics, providing genome-level resolution for characterization and tracing rather than replacing frontline confirmatory testing. In recent years, nanopore sequencing has been increasingly adopted in veterinary medicine, reflecting its expanding role at the animal–human–environment interface emphasized by the One Health framework. This review integrates current nanopore sequencing strategies and applications for animal pathogen detection—across viral, bacterial, and parasitic systems—and places them in the context of outbreak response, surveillance, and genome-informed decision-making (Figure 1). We also examine current limitations and outline future directions that may facilitate wider and more standardized use of nanopore sequencing in veterinary diagnostics.

This manuscript provides a structured overview of the current evidence on nanopore sequencing for veterinary pathogen detection. We surveyed relevant English-language literature indexed in PubMed, Web of Science, and Scopus (2015–2025) using terms related to nanopore sequencing platforms, metagenomics, amplicon and adaptive sampling strategies, veterinary/animal hosts, pathogen detection, and antimicrobial resistance. We prioritized studies with direct veterinary relevance and supplemented them with widely cited methodological work from human clinical genomics when the workflow principles were transferable.

Accordingly, this review focuses on (i) technical principles and sequencing strategies, (ii) sample-to-answer workflow components from specimen processing to bioinformatics interpretation, (iii) representative applications across viral, bacterial (including antimicrobial resistance, AMR), and parasitic diagnostics, and (iv) comparisons with conventional methods, current limitations, and practical directions toward more standardized veterinary and One Health surveillance.

## 2. Technical Principles and Advantages

Recent advances in nanopore chemistry and deep-learning-based basecalling have markedly improved sequencing accuracy, and Q20-class reads are now achievable under optimized conditions, making consensus genomes increasingly robust for typing and surveillance [5]. With complementary error-correction and polishing tools, nanopore data are also becoming more reliable for variant detection, supporting broader use in veterinary diagnostics.

### 2.1. Nanopore Sequencing Mechanism

A nanopore sequencing platform exploits nanoscale protein pores embedded in a membrane to sequence nucleic acids by measuring changes in ionic current as single DNA or RNA molecules pass through the pore [6]. This single-molecule, label-free sequencing method fundamentally differs from second-generation “sequencing-by-synthesis” techniques and eliminates the need for PCR amplification in some protocols, thereby avoiding biases and enabling direct epigenetic or RNA reading. To translate this sensing mechanism into actionable veterinary diagnostics, the overall performance of nanopore sequencing depends on upstream sample processing and downstream analytical choices. In the following section, we therefore summarize practical “sample-to-answer” workflows and sequencing strategies that connect specimen handling, library preparation, and interpretation.

### 2.2. Real-Time and Long-Read Data

A key advantage of nanopore sequencing is its real-time data generation. Basecalling begins as soon as nucleic acids pass through the pores, enabling preliminary pathogen identification within minutes—an important feature during outbreaks or clinical emergencies. This continuous data flow also supports adaptive sequencing, such as ONT’s adaptive sampling, which selectively enriches target DNA in real time and has been shown to improve *Plasmodium* detection directly from blood samples [7].

Nanopore platforms also produce ultra-long reads, far exceeding those from Sanger or NGS. Reads tens to hundreds of kilobases in length facilitate de novo genome assembly by spanning repetitive regions and resolving structural variants, often yielding closed bacterial genomes or high-quality hybrid assemblies. Recent studies further show that ONT-only assemblies of genomes can approach the contiguity and completeness of hybrid references, reflecting improvements in nanopore chemistry and basecalling accuracy [8].

### 2.3. Portability and Field Deployment

The portability of nanopore sequencers is another major advantage for animal pathogen detection. The hand-held MinION operates from a laptop and even battery power, enabling sequencing directly at farms, clinics, or field sites without relying on central laboratories. This on-site capability shortens diagnostic turnaround times and avoids delays or degradation associated with sample transport. In avian influenza surveillance, for example, field-based nanopore sequencing allowed immediate subtype identification from wild bird samples, eliminating multi-day waits for laboratory processing [9]. In addition, MinION-based short amplicon sequencing has been deployed during transboundary animal disease outbreaks in Mongolia, allowing rapid identification and genotyping of African swine fever virus (ASFV), classical swine fever virus (CSFV), and foot-and-mouth disease virus (FMDV), with actionable results generated within the same day of sample collection [4]. Together, these studies demonstrate the robustness of nanopore sequencing workflows in non-traditional environments and highlight their value for real-time, point-of-need pathogen genomics in veterinary disease surveillance and outbreak response.

## 3. Nanopore Sequencing Strategies for Pathogen Detection

Building on the nanopore sensing principle described in Section 2.1, the real-world performance of nanopore sequencing in veterinary diagnostics is shaped by two coupled decisions: how the sample is processed and which sequencing strategy is selected. These upstream choices determine the amount and integrity of pathogen nucleic acids relative to host/background material, and therefore directly affect read yield and quality, taxonomic resolution, and the reliability of downstream interpretation. Accordingly, this section summarizes workflow-oriented “sample-to-answer” approaches that are commonly used for animal pathogen detection and surveillance.

**Figure 2:**
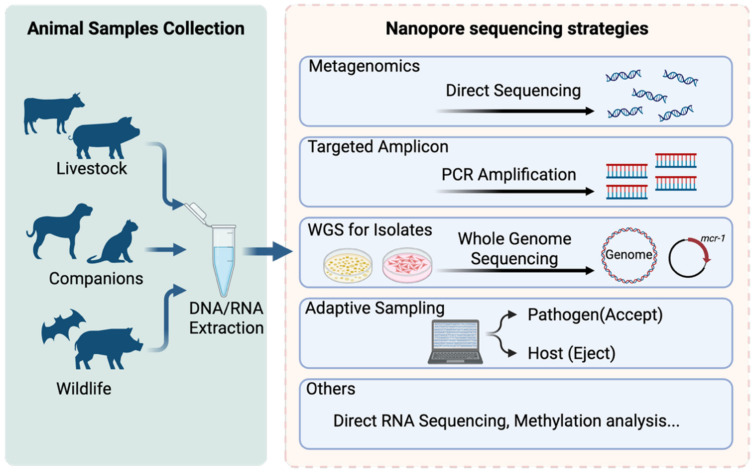
Schematic overview of nanopore sequencing strategies for animal pathogen detection. Animal-derived samples from livestock, companion animals, and wildlife are processed for DNA/RNA extraction and analyzed using multiple nanopore-based strategies. These include untargeted metagenomic sequencing, targeted amplicon sequencing, whole-genome sequencing of isolates, and adaptive sampling for host depletion or pathogen enrichment. Additional applications such as direct RNA sequencing and methylation analysis further expand the analytical scope of nanopore platforms in veterinary diagnostics and surveillance.

In practice, nanopore-based detection strategies can be broadly grouped into (i) metagenomic sequencing, (ii) targeted amplicon sequencing, (iii) isolate-based whole-genome sequencing, and (iv) adaptive sampling. These approaches are not mutually exclusive in veterinary medicine and are often combined depending on pathogen biology, sample matrix, and the clinical or surveillance objective. A schematic overview of these strategies is provided in Figure 2.

Regardless of strategy, several pre-analytical variables can dominate diagnostic sensitivity and interpretability, especially for host-rich or inhibitor-rich veterinary matrices (e.g., milk, tissues, feces). Key factors include nucleic acid extraction efficiency and inhibitor removal, the relative proportion of host/background nucleic acids, fragmentation and input quantity/quality, and library preparation choices (e.g., ligation vs. rapid kits, multiplexing and barcode crosstalk). Where feasible, the inclusion of negative controls and matrix-matched controls is important to contextualize low-abundance detections and reduce false positives during downstream classification and AMR/virulence annotation.

### 3.1. Targeted Amplicon Sequencing

Targeted amplicon sequencing uses PCR or RT-PCR to enrich predefined genomic regions before nanopore sequencing and is widely applied for rapid detection and genotyping of known animal pathogens. In veterinary medicine, this approach is most implemented using single-pathogen or tiled amplicon designs, providing high analytical sensitivity and short turnaround times. For example, a long-amplicon tiling PCR approach on the MinION was used to rapidly obtain the ~15 kb whole genome of Newcastle disease virus from poultry fecal samples [10]. In that study, overlapping PCR fragments covering the entire NDV genome were sequenced and assembled within hours, enabling near real-time characterization of the outbreak strain. Similarly, researchers have developed amplicon sequencing workflows for avian influenza viruses that amplify all eight gene segments, allowing full-genome sequencing of influenza A directly from field samples [9]. These applications primarily rely on well-characterized targets and curated primer sets, ensuring robustness and reproducibility in veterinary diagnostic and reference laboratories.

In addition to single-pathogen designs, multiplex amplicon panels targeting multiple candidate pathogens or pathogen groups have been developed to support etiological investigation when disease causation is initially unclear. These approaches aim to distinguish among likely viral or bacterial causes of syndromic presentations by simultaneously amplifying several predefined targets. While many multiplex or broad-range amplicon panels have been developed and validated in human diagnostics or implemented using second-generation sequencing platforms, recent veterinary studies have demonstrated the feasibility of multi-pathogen targeted nanopore sequencing in outbreak settings [11]. For example, a MinION short-amplicon workflow was used to rapidly identify and genotype multiple transboundary animal disease viruses (ASFV, CSFV, and FMDV) during outbreaks in Mongolia, delivering actionable sequence-based typing within the same working day [4]. Together, these studies indicate that multiplex amplicon-based nanopore sequencing can bridge the gap between highly specific PCR assays and untargeted metagenomic sequencing, and represents a promising direction for etiological diagnosis and syndromic surveillance in veterinary medicine.

### 3.2. Whole-Genome Sequencing for Isolates or Samples

Whole-genome sequencing involves nanopore sequencing of cultured pathogens or enriched material to obtain comprehensive genomic information. In veterinary bacteriology, this approach is central to high-resolution pathogen characterization, including identification of virulence factors, antimicrobial resistance genes, and mobile genetic elements [12]. Nanopore long reads enable assembly of near-complete bacterial chromosomes and plasmids, providing insights into the genomic context and potential transmission of resistance determinants. Although culture requirements can extend overall turnaround time, isolate-based whole-genome sequencing (WGS) remains essential for detailed epidemiological analysis and One Health investigations.

For isolate-based nanopore WGS in veterinary bacteriology, a practical analysis workflow can be employed whereby raw signal files are first subjected to basecalling and quality control as described above. Long-read de novo assembly is then performed—commonly with Flye [13] for bacterial genomes—to reconstruct chromosomes and frequently near-complete plasmids, followed by polishing using Racon and Medaka to improve consensus accuracy. Where short-read (Illumina) data are available, hybrid approaches can further improve base-level accuracy and reduce residual small errors: for example, true hybrid assembly with Unicycler [14] leverages the accuracy of short reads together with the structural resolving power of long reads to produce complete bacterial genomes, and short-read-based polishing with Pilon [15] can be used to correct remaining base errors and small indels in long-read assemblies to obtain highly accurate finished genomes. Assembly quality is assessed using QUAST [16], and the polished assembly is then annotated with Prokka [17] or RAST [18] to generate standardized gene calls for downstream interpretation. For AMR-focused outputs, resistance determinants are identified with AMRFinderPlus. Finally, for epidemiology investigations, isolates can be placed into gene-by-gene typing (cgMLST/wgMLST) using chewBBACA [19] and Roary [20], and phylogenetic context can be generated by aligning core loci/whole genomes with MAFFT and inferring trees with FastTree, with optional reference-based SNP confirmation again relying on Snippy (https://github.com/tseemann/snippy) (accessed on 25 December 2025) where appropriate.

### 3.3. Metagenomic Sequencing

Nanopore-based metagenomic sequencing enables untargeted analysis of all nucleic acids present in a sample and is particularly well suited for situations where the causative agent is unknown or multiple pathogens may be involved. In veterinary medicine, this approach supports broad-spectrum detection of viruses, bacteria, and parasites directly from clinical or environmental samples, with real-time data generation allowing early identification during sequencing [3]. The long-read data provides additional genomic context, facilitating partial or complete genome reconstruction and interpretation of resistance or virulence genes. For example, metagenomic nanopore sequencing has been used to identify unexpected viral agents in respiratory or enteric disease outbreaks and to characterize complex pathogen communities in fecal or environmental samples [21].

**Figure 3:**
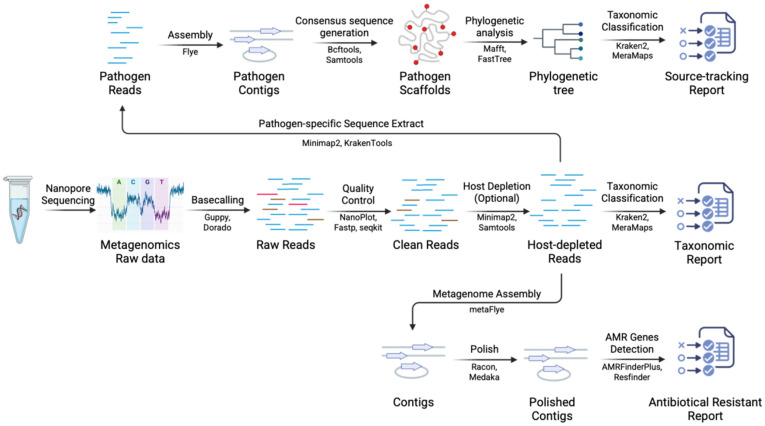
Representative nanopore long-read metagenomic analysis workflow for veterinary pathogen detection. Following DNA/RNA extraction and nanopore sequencing, data analysis proceeds through three optional modules. First, direct taxonomic profiling enables rapid species-level identification and community overview from quality-controlled, host-depleted reads. Second, in-depth analysis of specific pathogens is performed by extracting reads assigned to target organisms for genome assembly, polishing, and phylogenetic or source-tracking analyses. Third, antimicrobial resistance (AMR) analysis links resistance genes to reads or assembled contigs, enabling characterization of resistance determinants within clinical or environmental samples.

A practical nanopore long-read metagenomic workflow is summarized in Figure 3, and it typically proceeds as follows: (i) basecalling and (if multiplexed) demultiplexing, commonly performed with ONT’s Guppy or Dorado (https://github.com/nanoporetech/dorado) (accessed on 25 December 2025); (ii) read-level quality control (QC) and filtering, where long-read QC suites such as NanoPlot/NanoPack [22], fastp [23], seqkit [24] and simple length/quality filters are widely used to summarize and standardize input read sets; (iii) host read depletion for host-rich veterinary matrices (milk, tissues, respiratory samples) by mapping reads to the host reference using long-read aligners such as minimap2 [25] (often followed by basic BAM/FASTQ handling with samtools [26]) and removing host-mapped reads before microbial inference; (iv) taxonomic classification and abundance profiling, where fast k-mer classifiers such as Kraken2 [27] are commonly used for rapid screening, while long-read-aware mapping-based classifiers such as MetaMaps [28] are used when information-rich, strain-aware assignments are preferred; (v) optional genome-resolved assembly when the goal includes reconstructing pathogen genomes and linking virulence/AMR determinants to genomic context, using long-read metagenome assemblers such as metaFlye [29], followed by consensus polishing with tools such as Racon [30] and Medaka (https://github.com/nanoporetech/medaka) (accessed on 25 December 2025) to improve contig accuracy; and (vi) functional interpretation, particularly AMR gene detection from reads/contigs/assemblies using curated resources such as NCBI AMRFinderPlus [31] and ResFinder [32]. Finally, for pathogen-specific downstream analyses, reads assigned to a target pathogen can be extracted based on mapping/classification results and used for reference-guided consensus assembly and genomic epidemiology. Briefly, reads are mapped to an appropriate reference (e.g., using minimap2 or KrakenTools), a consensus genome is generated, and phylogenetic placement and outbreak source tracking can then be performed using established toolchains such as MAFFT [33] and FastTree [34].

### 3.4. Adaptive Sampling

Adaptive sampling is a real-time selective sequencing strategy unique to nanopore platforms, allowing reads to be accepted or rejected during sequencing based on partial sequence alignment to predefined references [35]. In pathogen detection, adaptive sampling is primarily applied as a software-based enrichment approach to reduce host DNA background or to preferentially sequence pathogen genomes in host-rich samples. For example, selective nanopore sequencing has been applied to enrich *Plasmodium* DNA from human blood samples, enabling recovery of parasite genomes and monitoring of resistance-associated loci despite overwhelming host DNA background [35]. Although many adaptive sampling studies have been conducted in human clinical contexts, the underlying principles are directly applicable to veterinary samples [36]. These studies highlight adaptive sampling as a promising adjunct to nanopore metagenomics, particularly for low-biomass infections and field-deployable veterinary diagnostics.

## 4. Applications in Viral Pathogen Detection

Nanopore sequencing has significantly advanced animal virology by enabling rapid whole-genome sequencing and molecular epidemiology in both outbreak response and routine surveillance. Because many animal viruses spread rapidly and require timely strain or subtype identification to guide control measures, the speed and field deployability of nanopore sequencing have proven especially valuable. Its use has been repeatedly demonstrated across diverse viral pathogens, supporting real-time decision-making in veterinary settings.

### 4.1. Rapid Outbreak Identification

Portable nanopore sequencing is particularly valuable during outbreaks of transboundary animal diseases, where same-day confirmation and actionable typing are needed to guide control measures. A representative example is MinION short-amplicon sequencing applied to outbreak-derived tissues in Mongolia, which enabled rapid identification and sequence-based typing of ASFV, CSFV, and FMDV within a single working day, supporting timely outbreak decisions [4].

Avian influenza investigations have similarly benefited from rapid nanopore workflows that couple PCR with long-read sequencing to generate subtype/pathotype information and segment-level genomes on short timelines. For example, an on-site compatible approach demonstrated that influenza A virus genomes could be sequenced and characterized with diagnostic utility in <24 h, providing an operational bridge between initial screening and genomics-informed outbreak response [37].

### 4.2. Routine Surveillance and Real-Time Monitoring

Nanopore sequencing is increasingly used for routine surveillance to generate timely whole-genome data for molecular epidemiology, including lineage tracking, geographic spread, and antigenic drift relevant to vaccine updates. A well-known example is influenza surveillance at the swine–human interface, where on-site nanopore sequencing and rapid analytics enabled interpretation of 13 swine influenza virus genomes within ~18 h, providing near–real-time genomic evidence relevant to risk assessment and vaccine strain considerations [9].

For avian influenza surveillance, nanopore workflows have also been adapted to field-like sample types and surveillance materials. For instance, sequencing approaches targeting HA/NA from wild bird feces using nanopore (including Flongle-based implementations) have been reported with accuracy comparable to conventional methods, supporting scalable integration of genomics into routine monitoring programs [38].

## 5. Applications in Bacterial Pathogen Detection and AMR Monitoring

Advances in nanopore chemistry and basecalling algorithms have substantially improved long-read accuracy, and recent studies show that, for many microbial genomes, ONT-only assemblies can approach the contiguity and completeness of hybrid references. This has important implications for veterinary AMR surveillance, as near-finished genomes and plasmids can increasingly be obtained using nanopore data alone, enabling more accurate characterization of resistance determinants and their genomic context [8,39]. In veterinary microbiology, timely identification of bacterial agents and their resistance determinants is crucial for guiding treatment decisions and controlling outbreaks in both livestock and zoonotic settings. Conventional bacteriological workflows, such as culture-based identification, biochemical assays, and targeted PCR, can be slow and often provide only partial information. By contrast, nanopore sequencing offers a complementary and, in many cases, more comprehensive approach by enabling rapid acquisition of genomic data, including resistance genes and virulence factors, directly from clinical samples or mixed bacterial cultures [40].

### 5.1. Rapid Identification and Genome Assembly

ONT long reads enable rapid bacterial species identification and, critically, facilitate reconstruction of complete or near-complete genomes, including chromosomes and plasmids. Fully resolved replicons are increasingly important for interpreting bacterial epidemiology and for locating resistance determinants within their mobile genetic contexts—information that is often fragmented or ambiguous in short-read-only draft assemblies. Hybrid strategies (long reads for structure, short reads for polishing) remain a common best practice for achieving “finished” bacterial genomes, but improvements in nanopore chemistry and basecalling have substantially increased the feasibility of high-contiguity ONT-only assemblies in many settings [5].

Evidence for high-quality long-read assemblies includes studies demonstrating near-finished microbial genomes from isolates or even metagenomes without short-read or reference polishing under optimized conditions [8]. In applied pathogen genomics, nanopore sequencing has also been used to generate complete chromosomes and plasmids rapidly, supporting genomic characterization of foodborne and zoonotic bacteria [41]. In veterinary and animal-associated contexts, long-read sequencing (often with hybrid assembly) has been used to close genomes from poultry and production-chain isolates—such as complete circular Salmonella genomes from poultry production and closed genomes from broiler farms/processing environments—providing improved resolution for AMR and source-tracking analyses [42].

### 5.2. Antimicrobial Resistance Gene Detection

Nanopore sequencing has become one of the most impactful approaches for AMR investigation in veterinary bacteriology because it can deliver genome-scale resistance information while simultaneously resolving the genetic context in which resistance determinants reside. Compared with phenotype-based antimicrobial susceptibility testing (AST) or targeted PCR panels, nanopore WGS supports comprehensive resistome profiling from isolates, including acquired resistance genes and, when coverage is sufficient, clinically relevant chromosomal mutations. As read accuracy and assembly performance continue to improve, nanopore data increasingly enable near-finished reconstructions that are more informative for transmission and epidemiological interpretation than fragmented short-read drafts [43].

A major strength of nanopore long reads is the ability to resolve plasmids and other mobile genetic elements that mediate AMR dissemination in animal-associated bacteria. For example, Li, Peng and colleagues used nanopore sequencing to elucidate the reorganization of large mcr-1–bearing MDR plasmids, highlighting how mobile elements can drive plasmid fusion/rearrangement and thereby alter the mobilization potential and co-selection patterns of resistance determinants [44]. In a related technical evaluation, Peng et al. showed that nanopore long-read sequencing can rapidly reconstruct complete genomes and complex resistance plasmids carrying clinically important resistance genes (e.g., *tet*(X), *tmexCD-toprJ*, *bla*_VIM-2_), reinforcing the value of long reads for mapping MDR architectures that are difficult to resolve with short reads alone [45].

Beyond cultured isolates, long-read metagenomic sequencing provides an increasingly practical route to study AMR in animal microbiomes and production environments by linking ARGs to their host taxa and mobile contexts. In poultry-associated fecal microbiomes, Peng et al. applied long-read metagenomics and demonstrated that prevalent ARGs are frequently shaped by plasmid carriage and mobile elements, with high-copy small plasmids contributing disproportionately to the observed resistome patterns—an insight that is difficult to obtain from short-read data alone [21]. Collectively, these developments indicate that nanopore sequencing is well positioned to support veterinary AMR surveillance by enabling both rapid resistance profiling and genome-context interpretation, particularly for plasmid-borne resistance and complex MDR regions.

### 5.3. Foodborne Pathogen Surveillance

Many bacterial pathogens originating from animals are also major foodborne hazards for humans (e.g., *Salmonella*, *Campylobacter*, Shiga toxin-producing *Escherichia coli*, and *Listeria*). Nanopore sequencing has therefore been explored for foodborne outbreak investigation and strain-level characterization in contexts where speed and genomic resolution are critical. A proof-of-concept study demonstrated strain-level metagenomics for foodborne outbreak investigations using MinION/Flongle on spiked food samples and benchmarked long-read workflows against short-read metagenomics [46]. Real-time nanopore sequencing has also been classically demonstrated in outbreak settings for *Salmonella*, illustrating how rapid turnaround can accelerate actionable genomics even before conventional workflows complete [47].

For *Salmonella*, MinION-based WGS has been evaluated for rapid confirmation and serotype classification in food-industry contexts, demonstrating the feasibility of multiplex nanopore sequencing as a cost-efficient, high-throughput tool for routine surveillance and incident response [48]. Similar “rapid sequencing-to-answer” paradigms were demonstrated in a hospital-associated *Salmonella* outbreak, where real-time nanopore sequencing produced reliable, actionable genomic information in less than half a day, illustrating the operational value of ONT-style workflows for time-sensitive epidemiological investigations [47].

Nanopore sequencing has also been applied to other priority foodborne bacteria. For *Campylobacter jejuni*, comparative studies have assessed MinION, MiSeq, and hybrid approaches for genome reconstruction, supporting its use for typing and genomic epidemiology in food safety settings [49]. In poultry production, portable nanopore workflows have been demonstrated for rapid on-site detection of *Campylobacter* from caecal samples within hours, highlighting their potential for decentralized monitoring closer to points of production [50]. Finally, ONT has been evaluated for genotyping and surveillance applications in *Listeria monocytogenes*, further supporting its role as a flexible platform for foodborne pathogen monitoring across the supply chain.

### 5.4. One Health and Environmental Monitoring

The accessibility, portability, and increasingly standardized analysis stacks of nanopore sequencing have lowered barriers for participation in genomic surveillance, allowing regional laboratories and smaller veterinary programs to generate actionable AMR data without relying exclusively on centralized sequencing hubs. This is particularly relevant in One Health contexts, where resistant bacteria and resistance determinants move across animal, human, and environmental interfaces. For example, an in-house, low-resource workflow based on Oxford Nanopore WGS was implemented to characterize multidrug-resistant Enterobacter cloacae complex isolates associated with dairy farm environments in Sri Lanka, explicitly positioning ONT as a transferable approach for AMR surveillance in resource-limited settings [51]. Portable power and field-compatible workflows further extend this decentralization potential, as demonstrated by fully off-grid sequencing deployments that illustrate the feasibility of generating genomic data in remote settings [52].

In environmental surveillance, long-read sequencing is increasingly used to profile resistant bacteria and ARGs in matrices that connect animal production systems to wider ecosystems, including wastewater, receiving waters, and agricultural water sources. Nanopore-based long-read metagenomics has been used to characterize resistome “intrusion” from treated wastewater into receiving water bodies and to highlight the role of plasmids and integrons in ARG persistence and dissemination [53]. Earlier work also demonstrated that MinION sequencing can quantify resistance genes in sewage-associated bacterial indicators with results broadly consistent with short-read approaches, supporting the operational feasibility of long-read AMR monitoring in complex environmental samples [54]. In food and environmental interfaces relevant to animal production, precision long-read metagenomics has been applied to detect and assemble Shiga toxin-producing *E. coli* from irrigation water following enrichment, illustrating how long reads can support both detection and genome-context resolution in surveillance-relevant watersheds [55].

Overall, nanopore sequencing is becoming a practical tool for bacterial diagnostics and AMR surveillance in veterinary medicine: it supports rapid species/strain characterization, increasingly complete genome and plasmid reconstruction, and improved interpretation of resistance determinants in their mobilizable contexts. While constraints remain—most notably sample preparation, host/background DNA, and bioinformatics capacity—real-world implementations in veterinary and environmental settings indicate these barriers are progressively diminishing as chemistries, workflows, and user-friendly pipelines mature [53].

## 6. Applications in Parasitic Pathogen Detection

While nanopore sequencing was initially adopted in veterinary infectious disease largely for viral and bacterial genomics, its utility for parasitic pathogens is now becoming increasingly evident. Parasitic infections (protozoa, helminths, and ectoparasites) remain a major constraint on livestock production, companion animal health, and wildlife conservation, yet routine diagnostics (microscopy and targeted PCR) can struggle with species-level discrimination, cryptic taxa, and mixed infections. Long-read nanopore workflows help address these gaps by supporting longer barcoding/typing markers, improving taxonomic resolution in complex parasite groups, and enabling “assumption-light” screening of broader pathogen communities when etiologies are unclear. For example, MinION long-amplicon sequencing of a 771-bp HSP70 marker improved Leishmania species identification and enabled detection of co-infections across clinical and reservoir samples [56]. More recently, an Oxford Nanopore 18S rRNA amplicon-based method was developed and validated for trypanosomatid detection and genotyping, demonstrating species- and lineage-level resolution and the ability to resolve co-infections in samples that included animals and vectors [57].

Beyond single-organism typing, nanopore long-read metabarcoding is emerging as a practical approach for characterizing parasite communities and detecting unexpected pathogens in field-relevant veterinary settings. A clear example is the use of MinION-based metabarcoding to holistically profile canine blood-borne pathogen communities, where long reads supported broad detection and helped reveal rare or unusual organisms in an under-surveyed region [58]. For helminth control, molecular surveillance of anthelmintic resistance is increasingly implemented via deep amplicon sequencing of resistance-associated loci and can be coupled with nemabiome-style community profiling; however, much of the published veterinary resistance surveillance has historically been platform-agnostic or short-read-dominated, and should be framed as a sequencing-enabled strategy that nanopore workflows can increasingly support as accuracy and throughput improve [59]. Finally, host-background DNA remains a key sensitivity bottleneck for parasite detection in blood/tissue metagenomics; adaptive nanopore sampling has been shown to enrich *Plasmodium* reads directly from blood and recover near-complete genomes, providing a strong proof-of-principle for applying real-time enrichment concepts to other host-dominated parasite diagnostics [35].

## 7. Comparison with Conventional Methods

Conventional diagnostic methods used in veterinary medicine—including microbial culture, microscopy, antigen-based assays (e.g., ELISA), PCR/qPCR, and Sanger sequencing—remain foundational in routine practice because they are operationally mature, are comparatively cost-effective, and often yield clear, target-specific answers when a suspected agent is known. However, these methods typically address narrow diagnostic questions per assay and may require sequential or parallel testing to achieve strain-level characterization or to infer antimicrobial resistance, particularly when mobile genetic elements drive AMR dissemination. Table 1 provides a scenario-based comparison, highlighting the best-fit use cases, strengths, and limitations of nanopore sequencing versus conventional diagnostic approaches. Practical guidance from veterinary diagnostic laboratory perspectives emphasizes that test performance and interpretation depend strongly on sample quality, timing, and the clinical question, and that combining complementary modalities is often necessary to strengthen diagnostic conclusions [60].

### 7.1. Advantages and Positioning of Nanopore Sequencing

Nanopore sequencing differs fundamentally by generating real-time long-read genomic data that can support genome-resolved typing, mixed-infection detection, and contextualization of AMR determinants (e.g., plasmid-borne resistance) within a single workflow when paired with appropriate bioinformatics. In practice, nanopore sequencing is best positioned as a complement rather than a replacement for conventional assays: rapid antigen/PCR testing can provide immediate case-level decisions, whereas sequencing adds higher-resolution characterization for outbreak tracing, surveillance, and One Health investigations [60,61].

### 7.2. Diagnostic Reliability and Interpretation

Diagnostic performance of nanopore sequencing can vary substantially with sample matrix, pathogen load, and workflow choices, and in low-load specimens PCR/qPCR may remain more sensitive for targeted detection. For example, in an evaluation of nanopore sequencing for *Mycoplasma bovis* identification from bovine respiratory tract samples, performance relative to real-time PCR differed by workflow and sampling format, illustrating that sensitivity can be lower in routine clinical specimens while specificity remains high [62]. Similarly, detection concordance between qPCR and nanopore sequencing for influenza D virus in bovine respiratory samples was influenced by analytical workflow and read assignment, underscoring the need for transparent interpretation criteria and evidence thresholds [63]. In practice, laboratories commonly interpret low-abundance detections using pragmatic criteria (e.g., minimum pathogen-assigned reads and/or genome coverage, negative controls, and consistency across replicate/library preparations), particularly for host-rich matrices where host/background nucleic acids can reduce effective sensitivity.

## 8. Limitations and Future Directions

Nanopore sequencing is now a credible platform for veterinary pathogen genomics, but routine diagnostic deployment is still constrained by accuracy requirements at fine variant resolution, contamination control (including barcode-related artefacts), bioinformatics and database dependencies, and practical trade-offs among cost, throughput, and sample quality. Many of these constraints are diminishing, but they remain central considerations for method selection and for how results are validated and reported in clinical and surveillance settings [64,65].

### 8.1. Sequencing Accuracy

Despite major gains in chemistry and basecalling [8], nanopore reads still exhibit context-specific error profiles, which can influence SNP/indel calling, minor-variant detection, and the interpretation of resistance-associated mutations when sequencing depth is limited. Recent studies demonstrate that modern R10.4 datasets—especially when coupled with appropriate assembly and polishing—can yield near-finished microbial genomes without Illumina polishing, and systematic evaluations indicate that high-accuracy (including duplex) modes can support robust bacterial genome reconstruction using nanopore-only data under suitable conditions. In practice, nanopore data are already well suited for consensus genomes, species/lineage assignment, and genome-resolved typing. For analyses that hinge on a small number of variants (e.g., fine-scale transmission inference or specific resistance mutations), results are most robust when laboratories define minimum coverage and QC thresholds in advance and apply conservative reporting criteria [65].

### 8.2. Barcode Contamination, Cross-Talk, and Run-to-Run Carryover

High-level multiplex barcoding reduces per-sample cost, but it also increases the risk of false positives—especially for low-biomass or low-titer samples. Controlled MinION multiplex studies show that a small but measurable fraction of reads can be assigned to the wrong barcode if demultiplexing filters are too permissive [66]. This is why barcode calling is not just a “formatting” step: it directly affects diagnostic specificity, and more advanced demultiplexing methods have been developed to reduce misassignment [66].

Another practical issue is carryover between runs. When flow cells are reused (or libraries are loaded sequentially), residual DNA from a previous run can persist even after washing and may appear as low-level reads in the next run [67]. For surveillance workflows that interpret low read counts as detection, this can be misleading, so carryover must be actively managed. In practice, robust multiplex workflows use negative controls, conservative demultiplexing thresholds, chimera filtering, and clear run-level rules for handling low-read detections rather than treating any single read as evidence of a true positive.

### 8.3. Host-Background DNA, Sample Quality Requirements, and Sensitivity in Complex Matrices

In many clinical samples, most nucleic acids come from the host rather than the pathogen. This reduces the fraction of pathogen reads in metagenomic sequencing and often forces deeper sequencing unless host DNA is removed or pathogens are enriched first. Human nanopore metagenomics studies show that host depletion can be critical for obtaining rapid, interpretable results, and the same limitation is common in veterinary samples such as milk, respiratory specimens, and tissues [68].

Veterinary work illustrates this clearly. In bovine mastitis milk, culture- and amplification-independent nanopore metagenomics relied on host DNA removal and bacterial enrichment to achieve rapid pathogen detection and AMR profiling within clinically useful timeframes [69]. In bovine respiratory samples, long-read metagenomics has been compared with culture and AST, showing that accuracy depends strongly on sample type, pathogen load, and analysis thresholds. Overall, sample prep and enrichment choices (targeted PCR, host depletion/enrichment, or selective sequencing) usually have a larger impact on sensitivity than sequencing run time [69].

### 8.4. Bioinformatics, Databases, and Interpretability

Nanopore sequencing can generate very detailed results, but the analysis and interpretation are not straightforward. To produce reliable diagnostic reports, laboratories need validated pipelines, well-curated reference databases (including veterinary pathogens), and clear rules for calling positives [70].

A practical challenge is that nanopore software and basecalling models are updated frequently, and these changes can affect performance. This makes results harder to reproduce across time and between laboratories unless pipelines are standardized and databases are managed carefully. In veterinary work, the problem can be worse because pathogens and hosts are diverse and local strains may be missing from public databases. For this reason, many groups are moving toward fixed analysis environments, snapshotting databases for reporting, and using validation panels designed for common veterinary sample types [64,65].

### 8.5. Cost, Throughput, and Workflow Economics

Nanopore platforms can be deployed with less infrastructure, but the cost per sample still depends on practical factors such as how many samples you multiplex per run, the flow cell and kit used, hands-on library prep time, computing capacity for basecalling/analysis, and how much host DNA is present [43]. As throughput increases, the main bottleneck in routine use often becomes sample processing, QC, and interpretation rather than sequencing output alone. In practice, this usually supports a tiered strategy: use PCR/ELISA for high-throughput screening, and reserve nanopore sequencing for cases where genome-level information is needed (strain typing, AMR context, outbreak tracing, or unexplained syndromes), instead of doing metagenomics on every sample [43,64].

### 8.6. Biosafety, Biosecurity, and Ethical Considerations

Biosafety and biosecurity are critical considerations when implementing nanopore sequencing for veterinary pathogens, particularly during outbreak investigations and in field-deployable workflows. While sequencing itself does not increase pathogenicity, upstream steps such as specimen collection, transport, nucleic acid extraction, and any manipulation of potentially infectious material require risk assessment, appropriate containment, trained personnel, and adherence to institutional and national guidelines. These requirements are especially important for high-consequence animal diseases and zoonotic agents, where occupational exposure and environmental release must be minimized. Guidance from international and national frameworks emphasizes protocol-driven risk assessment, appropriate containment practices, and management systems for biological risk in veterinary laboratories and related facilities.

Field-based sequencing adds practical challenges, including safe specimen handling in non-laboratory environments, maintaining clear separation between “wet” specimen-processing steps and downstream computational analysis, and ensuring secure sample chain-of-custody and reporting during emergency responses. Accordingly, routine adoption in veterinary diagnostic laboratories will benefit from standardized biosafety training, documented procedures for specimen handling/inactivation when appropriate, and quality management aligned with biorisk management principles.

### 8.7. Regulatory and Implementation Barriers in Routine Veterinary Diagnostics

Regulatory and practical barriers currently limit routine adoption of nanopore sequencing in veterinary diagnostic laboratories. Beyond analytical performance, implementation typically requires documented validation and ongoing quality management, as well as inter-laboratory reproducibility and proficiency testing. In addition, routine deployment may be constrained by staff training needs, the requirement for computational infrastructure and standardized bioinformatics pipelines, and the challenge of defining interpretation thresholds that remain robust across sample types and pathogen loads. Accordingly, in many settings nanopore sequencing is most readily implemented as a complementary method alongside established frontline assays such as PCR/qPCR, while broader regulatory acceptance will benefit from harmonized standards and external quality assessment frameworks.

### 8.8. Future Directions and Emerging Applications

Beyond improving speed and consensus accuracy, a key future direction for nanopore-based veterinary diagnostics is to move from “what is present” toward “how it functions,” by routinely extracting native RNA and modification (methylation) signals from the same long-read data. Direct RNA sequencing (DRS) eliminates reverse transcription and PCR for RNA molecules, reducing amplification bias and enabling long-range resolution of transcript structures, including overlapping and heterogeneous viral RNAs. Proof-of-concept studies have demonstrated DRS for RNA viruses relevant to animal health, including sequencing the coding-complete influenza A virus genome from avian egg-propagated material and revealing the transcriptional landscape of PRRSV using native RNA reads [71,72]. In coronaviruses, full-length DRS has been used to resolve complex viral RNA populations (including structural variants) and provides a basis for extending these approaches to veterinary coronaviruses where transcript diversity and recombination are epidemiologically important [73].

In parallel, nanopore signal data offer a route to epigenetic and epitranscriptomic information. For DNA, seminal work showed that nanopore current shifts can be used to detect cytosine methylation directly, and subsequent frameworks extended this concept to map multiple cytosine and adenine methylation types—including demonstrations in *E. coli*—without special library chemistry [74,75]. For RNA, method development and reviews now outline how DRS can, in principle, support detection of modified ribonucleotides in viral RNAs, although robust calling remains dependent on controls and careful computational modeling [76]. Looking ahead, incorporating DRS and modification-aware analysis into veterinary workflows could enable richer outbreak and surveillance outputs—linking genome sequence with transcript architecture and epigenetic state—particularly for RNA viruses with complex transcription strategies and for bacterial pathogens where methylation patterns may inform strain biology and mobile element dynamics [76].

## 9. Conclusions

Nanopore sequencing has progressed from a specialized field tool to a practical platform for veterinary pathogen genomics, combining portability, real-time data generation, and long-read resolution. Across viral, bacterial, and parasitic pathogens, it can deliver rapid etiological confirmation and richer genomic context than conventional single-target assays, supporting applications such as outbreak response, lineage and genotype assignment, and genome-resolved investigation of mobile genetic elements. In particular, long reads strengthen interpretation of antimicrobial resistance by enabling linkage of AMR determinants to plasmids and other genomic contexts that are often fragmented in short-read assemblies.

At the same time, nanopore sequencing is not a universal replacement for established diagnostics. Its performance and interpretability remain strongly dependent on sample type and workflow design. Host-background nucleic acids can dilute pathogen signal in metagenomic sequencing, requiring deliberate enrichment or depletion strategies for host-rich matrices. High-plex multiplexing improves throughput and cost-efficiency but introduces risks of barcode cross-talk and carryover that can generate false positives if contamination controls and conservative demultiplexing thresholds are not enforced. Equally important, clinically defensible deployment requires standardized, version-controlled bioinformatics pipelines, curated reference databases that adequately represent veterinary pathogens and regional diversity, and transparent calling criteria that specify minimum read support, breadth/depth thresholds, and rules for handling low-abundance detections.

In practice, the most robust near-term implementation is a tiered diagnostic model. Targeted assays (e.g., antigen tests or PCR/qPCR) provide scalable, low-cost screening and rapid case-level decisions, while nanopore sequencing adds value when genome context changes management—such as rapid strain characterization during farm outbreaks, high-resolution AMR surveillance with plasmid resolution, investigation of mixed infections or atypical syndromes, and One Health monitoring across farm–food–environment interfaces. Continued advances in chemistry and basecalling, contamination-aware multiplexing, selective sequencing and host depletion, and user-friendly validated analytics are expected to expand the range of nanopore-only applications that can be reported with high confidence. As these enabling components mature and standardization improves, nanopore sequencing is well positioned to become an integral complement to conventional diagnostics, strengthening veterinary disease control and genomic surveillance at the animal–human–environment interface.

## Figures and Tables

**Table 1:** Comparative performance of nanopore sequencing and conventional veterinary diagnostic methods.

Category	Nanopore Sequencing	Culture	Microscopy/Morphology	ELISA	PCR & qPCR	Sanger Sequencing
**Primary Output**	Whole-genome sequence	Viable isolate	Morphology of parasite	Presence/ absence of specific antigen	Presence/ absence of target gene; Ct values	Sequence of targeted short fragment
**Diagnostic Breadth**	Broad, detects multiple pathogens simultaneously	Culturable organisms	Organisms with distinctive morphology	Highly target-specific	Highly target-specific	Highly target-specific
**Turnaround Time**	Hours to 1 day	24 h to weeks	Minutes to hours	Minutes to hours	Hours	Hours to days
**Sensitivity**	High	Variable	Variable	Very high for intended target	Very high for intended target	Very high for sequenced region
**Specificity**	High	High	Variable	Variable	Very high for sequenced region	Very high for sequenced region
**Strain Typing / Genotyping**	High	Possible	Not applicable	Not applicable	Limited to targeted loci	Limited to targeted loci
**AMR Detection**	Comprehensive	Phenotypic only	Not applicable	Not applicable	Limited to targeted loci	Limited to targeted loci
**Mixed Infection Detection**	High	Low	Low	Detects only target antigen	Limited to targeted loci	Limited to targeted loci
**Field Deployability**	High	Low	Moderate	High	Moderate	Low
**Cost**	High	Low	Very low	Low	Low–moderate	Moderate
**Interpretability**	Requires analysis pipelines	Straightforward	Straightforward	Straightforward	Straightforward	Straightforward for targeted regions
**Main strengths**	Real-time, long reads, genome-level typing	Viable isolate, AST, standardized	Rapid screening, low equipment, parasites	High-throughput, serology, low cost	Sensitive/targeted, fast, quantitative	High accuracy, short targets, confirmation
**Recommended scenarios**	Outbreak investigation, unknown etiology, mixed infections, genome-informed tracing, variant surveillance, AMR/virulence detection	Phenotypic susceptibility, isolation for downstream characterization, viable organism confirmation	Routine targeted screening, rapid in-field screening	Serosurveillance, vaccination monitoring, post-shedding diagnosis, retrospective exposure	Targeted routine diagnosis, high-throughput screening, low-load detection, quantification, confirmatory testing	Amplicon confirmation, small-scale genotyping, SNP verification

## Data Availability

No new data were created or analyzed in this study. Data sharing is not applicable to this article.
